# PhosFox: a bioinformatics tool for peptide-level processing of LC-MS/MS-based phosphoproteomic data

**DOI:** 10.1186/1477-5956-12-36

**Published:** 2014-06-26

**Authors:** Sandra Söderholm, Petteri Hintsanen, Tiina Öhman, Tero Aittokallio, Tuula A Nyman

**Affiliations:** 1 Institute of Biotechnology, University of Helsinki, P.O. Box 65 (Viikinkaari 1), FI-00014 Helsinki, Finland; 2 Institute for Molecular Medicine Finland (FIMM), University of Helsinki, Helsinki, Finland

**Keywords:** Database searching, Phosphoproteomics, LC-MS/MS, Data processing and analysis

## Abstract

**Background:**

It is possible to identify thousands of phosphopeptides and –proteins in a single experiment with mass spectrometry-based phosphoproteomics. However, a current bottleneck is the downstream data analysis which is often laborious and requires a number of manual steps.

**Results:**

Toward automating the analysis steps, we have developed and implemented a software, PhosFox, which enables peptide-level processing of phosphoproteomic data generated by multiple protein identification search algorithms, including Mascot, Sequest, and Paragon, as well as cross-comparison of their identification results. The software supports both qualitative and quantitative phosphoproteomics studies, as well as multiple between-group comparisons. Importantly, PhosFox detects uniquely phosphorylated peptides and proteins in one sample compared to another. It also distinguishes differences in phosphorylation sites between phosphorylated proteins in different samples. Using two case study examples, a qualitative phosphoproteome dataset from human keratinocytes and a quantitative phosphoproteome dataset from rat kidney inner medulla, we demonstrate here how PhosFox facilitates an efficient and in-depth phosphoproteome data analysis. PhosFox was implemented in the Perl programming language and it can be run on most common operating systems. Due to its flexible interface and open source distribution, the users can easily incorporate the program into their MS data analysis workflows and extend the program with new features. PhosFox source code, implementation and user instructions are freely available from https://bitbucket.org/phintsan/phosfox.

**Conclusions:**

PhosFox facilitates efficient and more in-depth comparisons between phosphoproteins in case–control settings. The open source implementation is easily extendable to accommodate additional features for widespread application use cases.

## Background

The human proteome is estimated to include up to 500,000 phosphorylation sites [1], but only a fraction of the potential phosphorylation sites have been identified so far. The advances in phosphopeptide enrichment procedures and high-throughput mass spectrometry instrumentation have led to rapid development of MS-based phosphoproteomics during the last few years, and currently thousands of phosphorylation sites can be detected from a single sample. In MS-based phosphoproteomics, protein identification and phosphopeptide mapping relies on database search engines, including Mascot [2], Sequest [3], X!Tandem [4], OMSSA [5], Andromeda/Maxquant [6], and Paragon [7]. However, the user is often limited with the choice of search engine(s) to those that are compatible with the raw data from the MS-instrument used.

There are several software solutions and bioinformatic tools designed for managing and extracting information from phosphoproteomics experiments, such as ArMone [8], ProteoConnections [9], PhosphoSiteAnalyzer [10], and PeptideDepot [11]. Additionally, there are protein modification site localization algorithms which are integrated in search engines and interfaces, for example Mascot delta [12] and PhosphoRS [13]. While these software solutions can be successfully used in certain applications, to our knowledge, there are no software solutions for directly comparing phosphoproteomic results on the phosphopeptide level between multiple different database search engines and/or between stimulated versus control samples. We have previously developed a tool named Compid [14] to integrate and compare proteomics data from Mascot and Paragon, but this software does not take into account modifications, such as phosphorylation, and cannot thus distinguish between phosphorylated proteins and peptides or their non-phosphorylated counterparts.

To meet these limitations, we developed and implemented a software tool, PhosFox, which enables peptide-level processing of phosphoproteomic data generated by several protein identification search algorithms (including Mascot, Sequest, and Paragon), as well as between-algorithm comparisons and multiple between-group comparisons. Moreover, adding support for other post-translational modifications is possible with the current implementation of PhosFox, and to demonstrate this we have included the possibility to process also acetylation with PhosFox. The open source and efficient implementation is easily extendable to promote its wide application to large-scale phosphopeptide analyses.

## Results and Discussion

**Figure 1:**
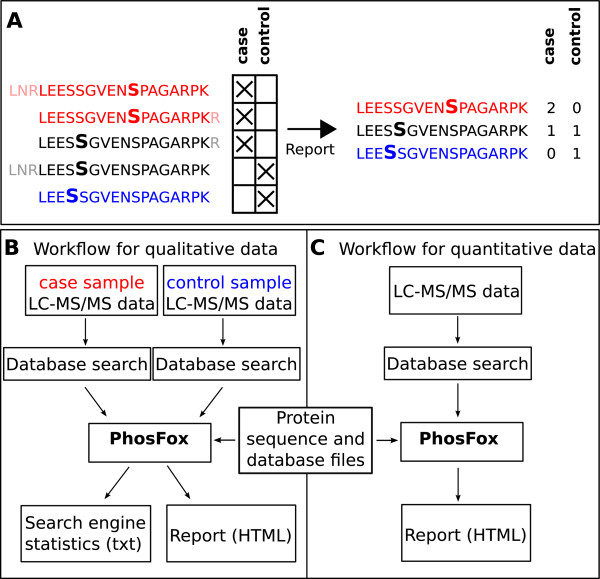
**An example of uniquely phosphorylated peptide (A) and workflows for qualitative and quantitative data processing (B, C).** The crosses mark the samples from which the corresponding peptides have been identified. The red peptides have a phosphoserine (bolded red S) that occurs only in the case sample: these peptides are deemed to be uniquely phosphorylated in the case sample. Note that the red peptides are considered as the same peptide in the report (counted as two) –despite having slightly different amino acid sequence lengths – because they have identical phosphosites. Their shared sequence is reported. The black peptides are not uniquely phosphorylated, because the same phosphosite (bolded black S) has been identified once in both the case and the control samples. Again, their shared sequence is reported. The single blue peptide is uniquely phosphorylated (bolded blue S) in the control sample.

**Figure 2:**
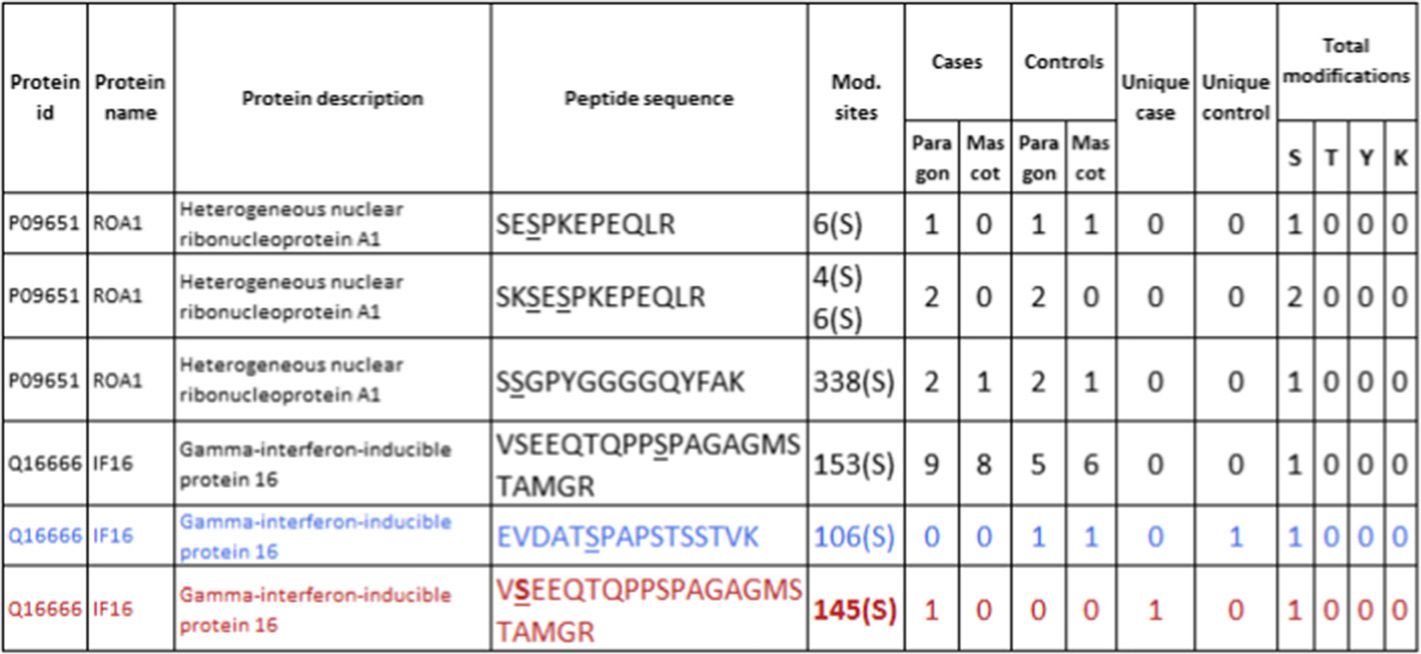
**An example of a PhosFox report.** The report contains: the protein accession number (id), the protein name and description, the peptide sequence with the modified amino acid(s) underlined, and the position of the modifications on the protein sequence, as well as further information whether the modification is novel (marked as bold). The following columns indicate in which sample(s) and by which search algorithm (in this example Paragon and Mascot) the peptide has been identified and whether it is unique to case or control. Additionally, the text color coding in the rows indicates whether the modified peptide is unique to case (red) or control (blue) or whether it is found in both case and control (black). The numbers of modified amino acids on the peptides are also listed in the output report. S = serine, T = threonine, Y = tyrosine, K = lysine.

In this work, we have created a new analysis tool, PhosFox, for processing and comparing phosphoproteomic data from multiple samples and several different database search algorithms. It is especially designed to find the phosphopeptide identifications from search engine results, and to distinguish uniquely phosphorylated peptides between different samples. Similarly to reporting phosphorylated peptides, PhosFox is also able to process acetylated peptides. The term ‘uniquely phosphorylated protein’ is used for describing a protein with at least one uniquely phosphorylated peptide, which has uniquely been matched to that particular protein in a particular sample. A ‘uniquely phosphorylated peptide’ is a phosphopeptide with a unique phosphorylation or phosphorylations either in the case or control sample (see Figure 1A for an example). This classification not only facilitates the discovery of differences in protein phosphorylation sites, but also improves the downstream analyses in the search of activated signaling pathways and networks. Workflows for qualitative and quantitative data processing are represented in Figure 1B and C. PhosFox allows the user to choose which datasets belong to the “control group” and which to the “case group”. In most quantitative experiments, the control and case groups have been combined before the database search step; in the case of iTRAQ [15] or SILAC [16] labeling, the samples have already been pooled before the LC-MS/MS analysis. An additional FASTA file containing protein sequences is needed for mapping the peptide sequences, and database files are required in order to identify modification sites described in the literature.The program outputs HTML reports with lists of phosphopeptides, including their phosphorylation differences between control and case groups, as well as between the database search engines. Additionally, the user can choose to produce a log file (as a plain text file) to report possible warnings. For instance, from this file the user sees if some of the input files have not been identified by the program, and if there are multiple matches for a certain peptide sequence with more than one protein included in the reference sequence database. An example of a standard PhosFox report can be seen in Figure 2. The PhosFox report contains: the protein id, the protein name and description, the peptide sequence with the modified amino acid(s) underlined, and the position of the modification site(s) on the protein sequence, as well as further information whether the modification site is described in the databases used or if it is “novel” (in that case the sites are in bold type). Additionally, the text color coding in the rows indicates whether the modified peptide is unique to case (red colored text) or control (blue colored text) or whether it is found in both case and control (black colored text). The numbers of modified amino acids (serines, threonines, tyrosines, and lysines) on the peptide are also listed in the output report. The search engine statistics include search engine-specific files of the uniquely modified proteins and all modified peptides identified in the samples.

**Figure 3:**
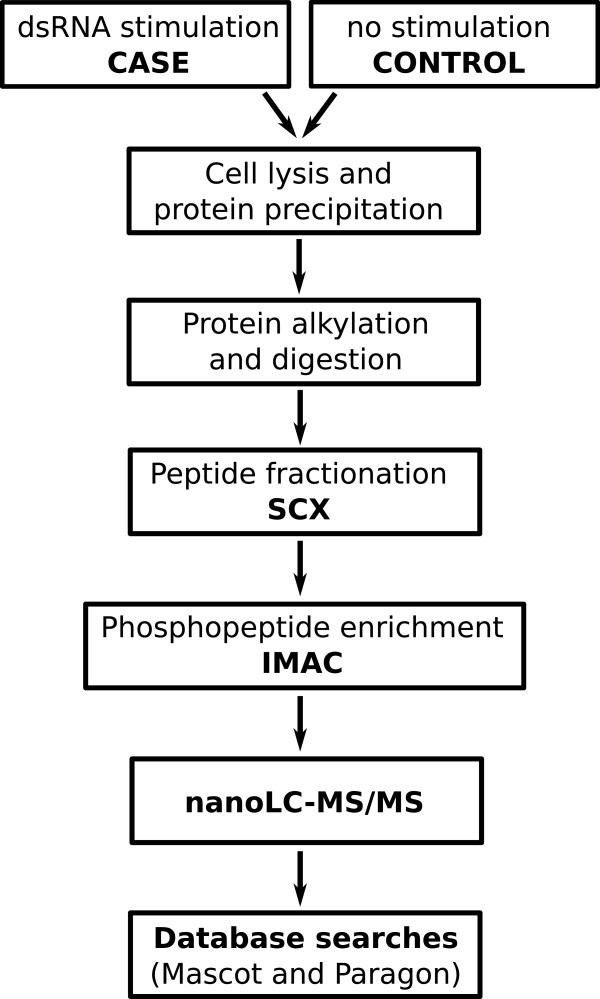
Experimental workflow for the preparation of the qualitative phosphoproteomics samples.

**Figure 4:**
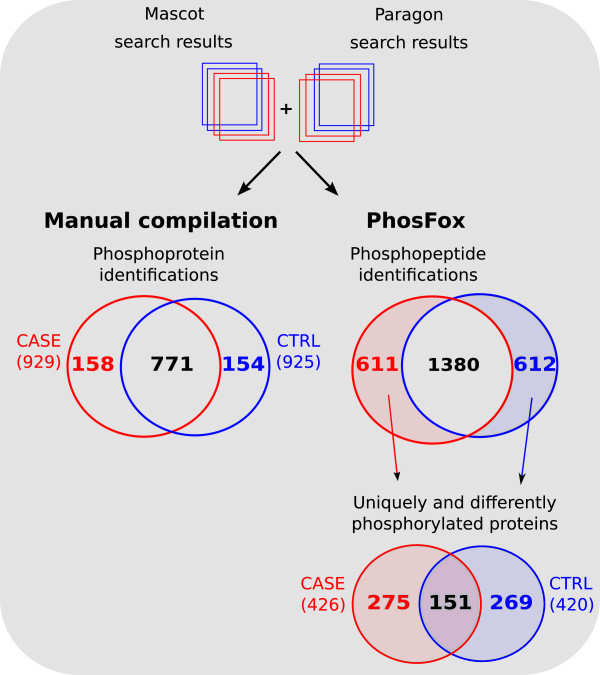
**Phosphoprotein and -peptide identification results from the qualitative phosphoproteomics samples after manual compilation of the database search results, as well as after automatic processing with PhosFox.** With manual compilation, 158 unique phosphoproteins for the case sample, and 154 unique phosphoproteins for the control sample were identified. With PhosFox, 611 unique phosphopeptides for the case sample, and 612 unique phosphopeptides for the control sample were identified, and 1380 phosphopeptides were identified from both control and case samples. The identical 1380 phosphopeptides across the control and case samples were discarded for further processing. The unique phosphopeptides resulted in identification of 426 unique phosphoproteins from the case, and 420 unique phosphoproteins from the control sample. From these, 151 proteins were identified in both samples with differences in phosphorylation sites.

As the first case study example of the use of PhosFox, we analyzed and compared two phosphoproteome datasets from human keratinocytes; an untreated control sample and a case sample transfected with a polyinosinic-polycytidylic acid (poly I:C) which mimics viral dsRNA-infection resulting in pro-inflammatory responses and apoptosis in human keratinocytes, responses characteristic for viral infection [17]. The experimental workflow is based on a previously published protocol [18] and is described in Figure 3. Briefly, the phosphopeptides were fractionated and enriched with strong cation exchange chromatography (SCX) combined with immobilized metal affinity chromatography (IMAC) before nanoLC-MS/MS analysis and database searches. Two biological replicates were analyzed, and the raw MS-data was searched separately with the Mascot and Paragon database search algorithms. When the search results from Mascot and Paragon (see Additional file 1: Table S1 for the search results and Additional file 2: Table S2 for a summarized table of phosphorylated and unphosphorylated peptide spectral matches) were manually compiled with Microsoft Excel, a total of 925 phosphorylated proteins were identified in the control sample and 929 in the case sample (Figure 4). The number of unique phosphoproteins was 154 in the control and 158 in the case sample. The 771 phosphoproteins that were identified from both samples include identically phosphorylated proteins, but also proteins with different phosphorylation profiles in control and case samples, and finding the phosphopeptide-level differences for these proteins with manual compilation is very laborious and error-prone. When the same samples were processed with PhosFox, we identified a total of 2,605 different (non-redundant) phosphopeptides, with 2,532 phosphorylated serine (85.2%), 418 phosphorylated threonine (14.1%), 15 phosphorylated tyrosine (0.5%), and eight phosphorylated lysine (0.2%) residues (see Additional file 3: Table S3 for the PhosFox report). The relative abundances of the phosphorylated residues compare well to the published data [19,20]. Altogether, PhosFox identified 1,992 phosphopeptides from the control and 1,991 from the case sample, with 1,380 phosphopeptides being identical between the samples. The identical phosphopeptides across the samples were not chosen for further processing and biological interpretation. The 612 unique phosphopeptides in the control sample (not found in the case sample) were linked to 420 proteins and the 611 unique phosphopeptides in the case sample (not found in the control sample) to 426 proteins. The identified peptides and their phosphorylation sites, for each sample and search engine separately, are included in Additional file 4: Table S4. The unique phosphoproteins identified from the control and case samples by Mascot and Paragon, are shown in Additional file 5: Table S5. In total 13 different acetylated peptides were also identified from the samples (see Additional file 3: Table S3 for the PhosFox report of the acetylated peptides). The Mascot and Paragon confidence scores for these acetylated peptides are shown in Additional file 6: Table S6. As a second case study example, we analyzed a previously published quantitative phosphoproteomic data from rat kidney inner medulla [21]. Similarly to the first case study example, the results show the advantage of using PhosFox compared to manual compilation (Additional file 7: Figure S1).

The results from the phosphoproteomic analyses can subsequently be analyzed for any associations with existing molecular information. Bioinformatic tools such as Ingenuity Systems Pathway Analysis (IPA) (https://analysis.ingenuity.com), Cytoscape [22] (http://www.cytoscape.org), or ExPlain (http://www.biobase-international.com) can be used for signaling network and pathway analyses. These downstream analyses usually rely on protein-level information, such as protein access numbers or gene names. Here, we used IPA for mapping proteins onto existing networks and pathways and classifying the proteins based on gene ontology (GO) annotations as well as KEA, a kinase enrichment analysis tool [23], to gain biological insight into the phosphoproteome data from our HaCaT keratinocyte experiments. The IPA analysis results of the manually processed datasets as well as the PhosFox-processed datasets are shown in Table 1. The identified canonical pathways and networks are different in the manually compiled case and control datasets compared to their PhosFox-processed counterparts. This indicates that the two ways of processing the phosphoproteomic data leads to different biological interpretation. The PhosFox-processed phosphoproteomic data includes detailed information of changes in the phosphorylation status of proteins in a sample compared to another. On the other hand, manual comparison, which is performed at the protein level, provides only information about which proteins are phosphorylated or not in one sample compared to another.

**Table 1:** Top-ranked canonical pathways and networks after dsRNA-stimulation of human keratinocytes and control samples

**A.**			
**Manual compilation**		**PhosFox**	
**Pathways**	**p-value**		**p-value**
Role of BRCA1 in DNA damage	0.0018	DNA methylation and transcriptional repression signaling	0.0012
Cell cycle: G2/M DNA damage checkpoint regulation	0.0061	Cyclins and cell cycle regulation	0.0027
Mismatch repair in eukaryotes	0.0082	Role of BRCA1 in DNA damage	0.0031
Phosphatidylethanolamine biosynthesis III	0.0087	Endometrial cancer signaling	0.0085
DNA damage-induced 14-3-3σ signaling	0.012	ATM signaling	0.013
**Networks**	**score**		**score**
Cell death and survival, cell cycle, nervous system development and function	21	Cellular assembly and organization, cellular compromise, cell death and survival	36
Cell cycle, DNA replication, recombination and repair, cell death and survival	19	Gene expression, cell signaling, post-translational modification	34
RNA post-transcriptional modification, cell morphology, cellular compromise	19	Cell cycle, cellular movement, gene expression	17
**B.**			
**Manual compilation**		**PhosFox**	
**Pathways**	**p-value**		**p-value**
ATM signaling	1.25E-04	Epithelial adherens junction signaling	5.65E-06
GADD45 signaling	4.85E-04	Remodeling of epithelial adherens junctions	1.26E-04
DNA damage-induced 14-3-3σ signaling	4.85E-04	Sertoli cell-sertoli cell junction signaling	1.78E-04
Role of CHK proteins in cell cycle checkpoint control	1.1E-03	ATM signaling	3.80E-04
Cell cycle: G2/M DNA damage checkpoint regulation	5.34E-03	Germ cell-sertoli cell junction signaling	8.43E-04
**Networks**	**score**		**score**
Cell cycle, DNA replication, recombination, and repair, gene expression	45	Cell morphology, cellular function and maintenance, cell cycle	39
RNA post-transcriptional modification, cell cycle, cellular movement	21	Cellular assembly and organization, DNA replication, recombination, and repair, cell morphology	37
Cellular development, cellular movement, connective tissue disorders	19	Organismal survival, organ morphology, respiratory system development and function	37

The IPA top-ranked canonical pathways and networks for the manually compiled and PhosFox-processed dsRNA-stimulated datasets **(A)** and control datasets **(B)** of the qualitative phosphoproteomic case study example.

**Figure 5:**
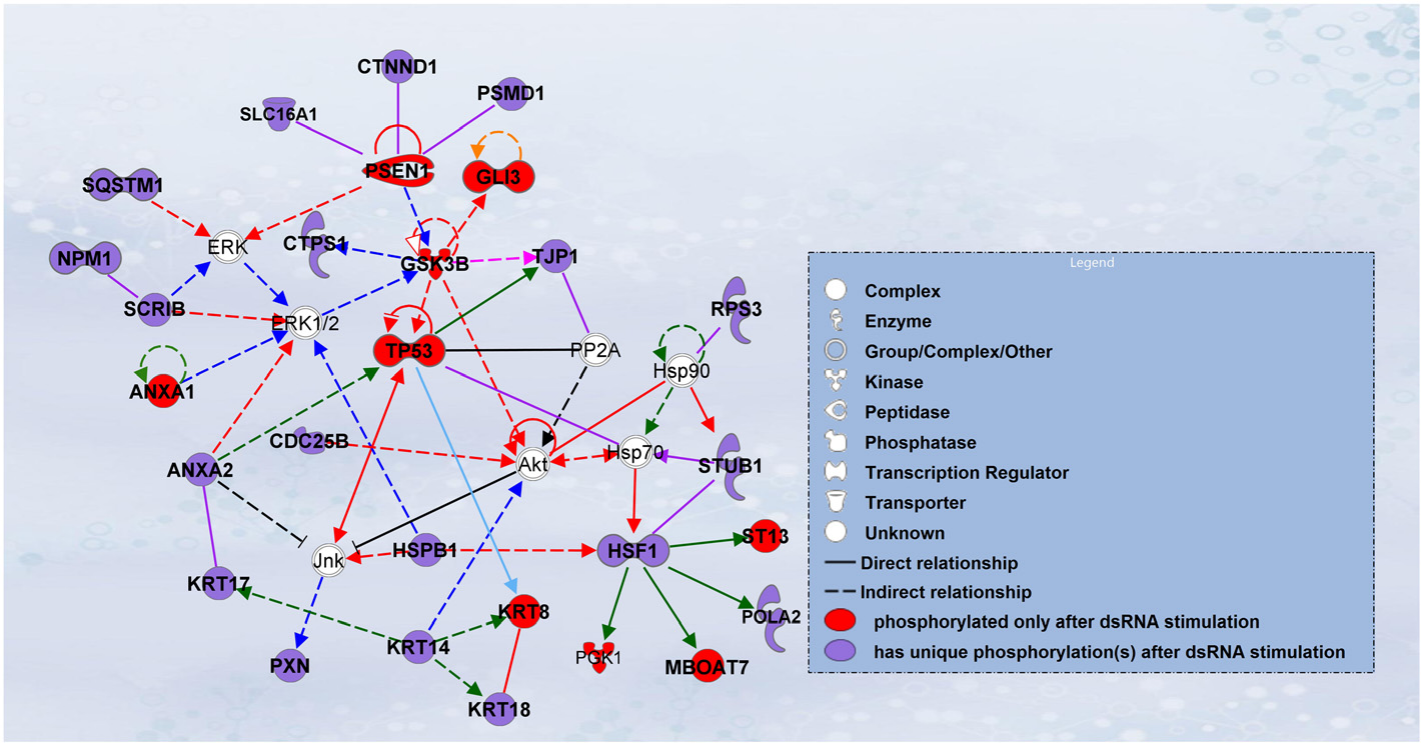
**The top-ranked IPA network of the PhosFox-processed case dataset of the qualitative phosphoproteomic case study example.** The most significant network from the PhosFox-processed case dataset (426 proteins) associated with cellular assembly and organization, cellular compromise, cell death and survival. The red nodes are proteins found to be phosphorylated only in the case sample (included in the manually compiled 158 unique phosphoproteins), whereas the violet nodes are proteins that have both common phosphopeptides in the control and case sample, as well as unique phosphopeptides in the case sample identified by PhosFox. The white nodes are included in the network through the IPA knowledge database and not found in the sample, but known to be in the network. The signaling networks were supported by at least one curated annotation from the Ingenuity Pathways Knowledge Base (Ingenuity® Systems, http://www.ingenuity.com). Solid and dotted lines indicate direct and indirect molecular interactions, respectively. The shape of the nodes indicates the molecular class. The color coding of the edges are: pink, regulation of binding; green, expression; red, activation; violet, protein-protein interactions; black, inhibition; orange, molecular cleavage; blue, phosphorylation/activation; light blue, transcription.

The top-ranked network identified from PhosFox-processed dsRNA-stimulated case dataset is associated with cellular assembly and organization, cellular compromise, cell death and survival (Table 1A and Figure 5). This network includes MAPK (ERK, ERK1/2 and Jnk) and PI3K/Akt signaling molecules which are directly or indirectly interacting with phosphoproteins detected from the case dataset. The MAPK and Akt family kinases were not directly found in the phosphoproteomic data, but these are known to be central players in many signaling pathways, and to target proteins regulating various cell processes. Their presence in the top-ranked network and their connections to proteins from the PhosFox-detected dsRNA-stimulated case dataset is therefore plausible. In contrast, the manually compiled case dataset lacks completely this top-ranked network and several of its interacting proteins. This further highlights that PhosFox has substantial impact on the results of the downstream analyses, and that the PhosFox processing significantly adds the amount of biological information that can be extracted from the phosphoproteome data.

Viruses are able to manipulate a variety of host-cell signal transduction pathways. The biological impact of the dsRNA stimulation versus no stimulation was studied in more detail with KEA, a kinase enrichment analysis tool [23]. The proteins with unique phosphorylation sites in the control and case samples were analyzed separately, and the best ranked kinases, kinase families, and kinase classes for these datasets are shown in Additional file 8: Table S7. Out of the 445 different kinases included in the KEA knowledgebase, substrates for 140 kinases were identified in the case dataset and substrates for 160 kinases in the control dataset. The dsRNA-stimulated dataset included more significantly enriched substrates for MAPK3 (ERK1) and MAPK8 (JNK1), compared to the control dataset. Moreover, substrates for kinases with known roles in regulation of infection were enriched in the case dataset, but not in the control dataset (p-value < 0.01). One of these kinases was protein kinase C beta type (PRKCB1), which is involved in immunity, apoptosis and NFκB signaling pathways [24]. Serine/threonine-protein kinase MARK1 is active in cell polarity, microtubule dynamics and Wnt signaling [25] and PRKDC (DNA-dependent protein kinase catalytic subunit) is known as a molecular sensor of DNA damage [26].

MAPK and PI3K/Akt signaling events regulate responses to extracellular stimuli including viral infections, but also various cellular activities such as cell metabolism and proliferation [27,28]. Cyclins and cell cycle regulation is included as one of the top-ranked pathways in the case dataset (Table 1A). Also other phosphoproteomic studies with focus on viral infections have demonstrated alterations in phosphorylation of proteins included in these signaling pathways [20,29]–[31], suggesting that these pathways have an important role in host-response against viral infection.

## Conclusions

At present, thousands of phosphopeptides and –proteins can be identified in a single experiment with high-throughput LC-MS/MS. However, a major difficulty in these studies is the downstream data analysis which is often laborious; in particular, the comparative analysis of the identified phosphopeptides between different samples and the comparison of identification results from different search engines often requires multiple, partially manual steps. To this end, we have developed PhosFox, which enables an automated and integrated phosphoproteomic data analysis. PhosFox compares phosphopeptide results generated with various database search engines across multiple sample groups, such as those with different treatments or time points. PhosFox supports both quantitative and qualitative phosphoproteomic data, and includes special features such as categorization of such phosphopeptides that are unique either to control or case group, or common to both groups. In conclusion, PhosFox facilitates efficient and more in-depth comparisons between phosphoproteins in case–control settings. The open source implementation is easily extendable to accommodate additional features for widespread application use cases, such as a motif-finding option, which would provide valuable information about the kinases that are phosphorylating the identified phosphorylation sites, leading to greater understanding of the functional impacts that these modifications have on cellular processes.

## Methods

### PhosFox software tool

The phosphopeptide data analysis program PhosFox was implemented in the Perl programming language. PhosFox is free software and can be run on most common operating systems, including Windows. Due to its flexible interface and open source distribution, the users can easily incorporate the program into their MS data analysis workflows and extend the program with new features. PhosFox source code, implementation and user instructions are available at https://bitbucket.org/phintsan/phosfox.

PhosFox has been designed so that the user can directly import the database search results as its input. The input data is imported in plain text format, either as comma-separated values (CSV) or tab-separated values (TSV). PhosFox supports the most common file formats and contents exported by the proteomic software being used for analyzing the MS results and performing the database searches. For example, the Mascot search results can directly be converted to CSV files. Paragon and Sequest generated peptide search results can be saved as TSV files. Moreover, if the database searches have been carried out through the Proteome Discoverer (Thermo Scientific) interface, the peptide spectral matches (PSMs) can be exported as plain text format files.

The user can import an arbitrary number of input files (peptide lists) for the analysis. Every input file is defined as either “case” (e.g. stimulated sample) or “control” (e.g. nonstimulated sample). If multiple search result files are added, the files are grouped and processed as one batch. The user can specify cut-off values for multiple quality scores, such as Mascot ion score or Paragon peptide confidence level. PhosFox does not test the confidence of phosphorylation assignments to particular amino acids in the peptide sequence matches, but the user can set a threshold for scores generated by modification site localization algorithms incorporated in search engines, such as Mascot delta [12] or PhosphoRS [13].PhosFox detects phosphorylated and acetylated amino acid residues for each peptide, which have been defined in the settings file (by default serine, threonine, tyrosine, and lysine), using the post-translational modification field in the corresponding input file. Non-phosphorylated and non-acetylated peptides are discarded from further processing. As a unique feature of the tool, each phosphorylated (or acetylated) peptide is examined whether it is uniquely modified in the case or the control sample (see Figure 1A for details). A ‘uniquely phosphorylated peptide’ is a phosphopeptide that has a unique phosphorylation or phosphorylations either in the case or control sample.

Peptides in quantitative datasets are treated similarly to peptides in qualitative sets: each peptide is checked for “enrichment” in either case or control sample by comparing the relative amount of detected peptides against a user-specified threshold. For example, the user can specify that if a phosphopeptide has more than a two-fold difference in the case sample, relative to the control sample, it is considered as enriched in the case sample. Such peptides are treated by PhosFox as if they were identified in a (qualitative) case sample. Similar strategy is used for identifying peptides enriched in the control sample.

In cases where multiple search engines are used, PhosFox can also compare similarities and differences between the results from the different search engines. Each input file is attributed to a specific search engine. If there are multiple search engines specified, a peptide is deemed uniquely phosphorylated in the case sample (resp. control) only if it has been detected in the case (control) sample by at least one search engine and not detected in the control (case) sample by any search engine. Furthermore, PhosFox divides unique phosphoproteins into different search engine-specific files, thus facilitating the extraction of either supporting or complementary information about identifications between the different search engines.

Finally, PhosFox reports novel phosphorylations (and acetylations) to the user by comparing the identified sites against those reported in the UniProt, PHOSIDA and PhosphoSitePlus databases. The program outputs HTML reports with lists of peptides, including their modification differences between control and case groups, as well as between the database search engines.

PhosFox is freely available online at: https://bitbucket.org/phintsan/phosfox. The homepage provides installation instructions and a user manual, and here it is possible to download and extract the distribution package, which includes all the required Perl modules. PhosFox is platform independent, but requires Perl version 5.6 or newer. This is already installed in most Unixes and unix-like operating systems (GNU/Linux, BSDs, OS X). For Microsoft Windows, we recommend Strawberry Perl, or the precompiled binary executable (see instructions on the homepage). Protein sequences in FASTA format are needed, and at the moment UniProt and NCBI RefSeq FASTA formats are supported. The detected peptide lists can be imported in plain text format (see the manual for details for supported file types), and a minimum of one case file and one control file is required. PhosFox can optionally detect acetylations and phosphorylations that have been described in the literature before. This feature is enabled by downloading and installing separate database files from the PhosFox web site. PhosFox is free software and requires no licensing either from academic or non-academic users. The source code can be redistributed and/or modified under the GNU General Public License or Artistic License.

### Qualitative phosphoproteome samples

Human keratinocytes, HaCaT cells (from ATCC) were transfected with 7 μg/ml dsRNA-analogue polyinosinic-polycytidylic acid (poly I:C) (Sigma-Aldrich) using Lipofectamine™ 2000 (Invitrogen) for 1 h or left untreated. The cells were collected and washed with PBS before they were lysed with HEPES lysis buffer (50 mM HEPES, 150 mM NaCl, 1 mM EDTA, 1% NP-40, pH 7.4) including protease and phosphatase inhibitor cocktails (Sigma-Aldrich). The cell lysates were centrifuged 11,686 × g for 15 min at 4°C and the protein content was measured with Bio-Rad DC™ protein assay (Bio-Rad). For the samples, 8 mg of protein was used. The proteins were precipitated with 10% TCA/acetone and resuspended in 1 ml of urea buffer (8 M urea, 400 mM NH4CO3, 20 mM DL-dithiotheitol, 1 mM EDTA, pH 8.5). The proteins were reduced, alkylated and enzymatically digested in-solution with lysyl endopeptidase (7.5 μg/sample, rLys-C Mass Spec Grade, Promega) for 2 hrs, which after the samples were diluted with 7 ml of destilled water, followed by digestion with trypsin (20 μg/sample, Sequencing Grade Modified Trypsin, Promega) for 16 hrs. Undigested proteins and cell debris were removed, and the samples were desalted on Sep-Pak Vac RP C18 cartridges (Waters). The peptides were fractionated by SCX-HPLC, using an ÄKTApurifier™ instrument (Amersham Biosciences). The peptides were separated on a 200 × 4.6 mm, 5 μm, 200 Å PolySULFOETHYL A™ column (PolyLC) by applying a gradient run with increasing salt concentration. The A buffer contained 10 mM KH2PO4, 20% acetonitrile, with a pH < 3. The gradient was set to 0–50% buffer B (buffer A + 0.4 M KCl) in 25 min, followed by 50–100% buffer B in 15 min. The flow rate was 1 ml/min and 1 ml fractions were collected by an autosampler. The SCX-fractions containing phosphopeptides were collected and desalted. Phosphopeptide enrichment was performed with IMAC using PHOS-Select™ Iron Affinity Gel (Sigma Aldrich) and SigmaPrep™ spin columns according to the manufacturer’s instruction. The enriched phosphopeptides were vacuum-dried and dissolved in 0.1% TFA, which after analyzed by nanoLC-MS/MS using an Ultimate 3000 nano-LC (Dionex) coupled to a QSTAR Elite hybrid quadrupole TOF-MS (Applied Biosystems/MDS Sciex) with nano-ESI ionization as previously described [17,32]. The samples were loaded on a ProteCol C18-Trap column (SGE) and separated on a PepMap C18 analytical column (15 cm × 75 μm, 5 μm, 100 Å) (LC Packings/Dionex) at 200 nl/min with a linear gradient of 0–40% acetonitrile in 120 min. The MS data was acquired with Analyst QS 2.0 software. Information-dependent acquisition method consisted of a 0.5 s TOF-MS survey scan of m/z 400–1400. From every survey scan two most abundant ions with charge states +2 to +4 were selected for product ion scans, and each selected target ion was dynamically excluded for 60 s. Smart IDA was activated with automatic collision energy and automatic MS/MS accumulation. The LC-MS/MS data were submitted through the ProteinPilot 4.0 interface (Applied Biosystems/MDS Sciex) to an in-house Mascot database search engine version 4.0 (Matrix Science), and to the ProteinPilot algorithm Paragon. The data were searched against the human canonical sequences in the Swiss-Prot database (version 01032013 with 539,616 sequences for the Mascot searches and version 01042013 with 539,829 sequences for the Paragon searches). Similar search criteria for both Mascot and Paragon were used and the criteria are listed, together with the original searches, as Additional file 1: Table S1. For additional confidence of the peptide identifications, the Mascot search results were filtered with an ion score expected cut-off value of 0.01, and the Paragon search results with a peptide confidence level of 99%. The raw data, together with the original Mascot and Paragon searches, has been deposited to the ProteomeXchange Consortium (http://proteomecentral.proteomexchange.org) via the PRIDE partner repository [33] with the dataset identifier PXD000577.

### Quantitative phosphoproteome samples

Published iTRAQ datasets [21] were used for the implementation and testing of the quantitative data support in PhosFox. As described in the original publication [21], rat inner medullary collecting duct samples were incubated with or without dDAVP, a V2 receptor-analog of vasopressin, at four different time points (0.5, 2, 5 and 15 min). The proteins were enzymatically digested and each peptide sample was labeled with 8-plex iTRAQ reagent. The labeled samples were combined into a single sample before SCX fractionation, Ga^3+^ IMAC, and LC-MS/MS analysis with a LTQ Orbitrap Velos mass spectrometer (Thermo Scientific). The MS/MS data was searched with the Sequest algorithm through the Proteome Discoverer platform (Thermo Scientific) on a concatenated database of the Rat Refseq Database (NCBI, March 3, 2010, 30,734 entries), and the abundance ratios (dDAVP/control) for the four time points were calculated. The 15 min time point from one of the three biological replicates was analyzed with PhosFox and compared to the original results (Additional file 7: Figure S1).

## Competing interests

The authors declare that they have no competing interests.

## Authors’ contributions

SS carried out the experimental case study, helped with the development of the software and drafted the manuscript. PH implemented the PhosFox application, wrote the code and helped to draft the manuscript. TÖ participated in developing PhosFox. TA and TN conceived and designed the experiments and wrote the manuscript. All authors read and approved the final manuscript.

## Supplementary Material

Additional file 1: Table S1 The database search results for the qualitative phosphoproteomic case study example. Click here for file

Additional file 2: Table S2 The number of unphosphorylated and phosphorylated peptide spectral matches (PSMs) of the qualitative phosphoproteomics case study example identified by Mascot and Paragon search engines in the different samples (control and case) and biological replicates (I-II). A threshold of 99% peptide confidence for the Paragon results and an ion score cut-off of 0.01 for the Mascot results were applied. Click here for file

Additional file 3: Table S3 The PhosFox reports of phosphorylated and acetylated peptides for the qualitative phosphoproteomic case study example. Click here for file

Additional file 4: Table S4 Results from the qualitative phosphoproteomic case study example processed with PhosFox: The Mascot and Paragon peptide and phosphosite identifications from the control and case samples. The phosphosite is indicated as the amino acid number on the total protein sequence and the type of amino acid carrying the phospho-group as a letter (S, T, Y or K). Click here for file

Additional file 5: Table S5 Results from the qualitative phosphoproteomic case study example processed with PhosFox: the unique phosphoproteins identified from the control and case samples by Mascot and Paragon. Click here for file

Additional file 6: Table S6 Mascot and Paragon confidence scores for the acetylated peptides identified by PhosFox in the qualitative phosphoproteomic case example. Click here for file

Additional file 7: Figure S1 The quantitative phosphoproteomic case study example. Previously published quantitative phosphoproteomic data from rat kidney inner medulla [21] of the 15 min time point from one of the three biological replicates was analyzed. The manually compiled search results are represented as a Venn diagram on the left and the PhosFox processed results as Venn diagrams on the right. Cutoff values of > 1.414 for the case peptides and < 0.707 for the control peptides were applied. With manual compilation, a total of 2,094 phosphoproteins for the case sample and 2,087 phosphoproteins for the control sample were identified. From these, 2,002 phosphoproteins were identical between the samples. With PhosFox, 325 unique phosphopeptides for the case sample and 344 unique phosphopeptides for the control sample were identified. In total, 4,025 phosphopeptides were identical between the samples. By taking into account the sample-unique phosphopeptides, 282 uniquely phosphorylated case proteins and 299 uniquely phosphorylated control proteins were identified. From these, 52 proteins had differences in phosphorylation sites between the case and control samples. Click here for file

Additional file 8: Table S7 The KEA kinase enrichment analysis results for the PhosFox-processed control and case datasets from the qualitative case study example. Click here for file
