# Supplementary material for: PhosFox: a bioinformatics tool for peptide-level processing of LC-MS/MS-based phosphoproteomic data
Source: Proteome Sci. 2014 Jun 26;12:36. doi: 10.1186/1477-5956-12-36 (PMC4098950; doi:10.1186/1477-5956-12-36)
Supplement: Additional file 7: Figure S1 — The quantitative phosphoproteomic case study example. Previously published quantitative phosphoproteomic data from rat kidney inner medulla [21] of the 15 min time point from one of the three biological replicates was analyzed. The manually compiled search results are represented as a Venn diagram on the left and the PhosFox processed results as Venn diagrams on the right. Cutoff values of > 1.414 for the case peptides and < 0.707 for the control peptides were applied. With manual compilation, a total of 2,094 phosphoproteins for the case sample and 2,087 phosphoproteins for the control sample were identified. From these, 2,002 phosphoproteins were identical between the samples. With PhosFox, 325 unique phosphopeptides for the case sample and 344 unique phosphopeptides for the control sample were identified. In total, 4,025 phosphopeptides were identical between the samples. By taking into account the sample-unique phosphopeptides, 282 uniquely phosphorylated case proteins and 299 uniquely phosphorylated control proteins were identified. From these, 52 proteins had differences in phosphorylation sites between the case and control samples. [file 1477-5956-12-36-S7.pdf]

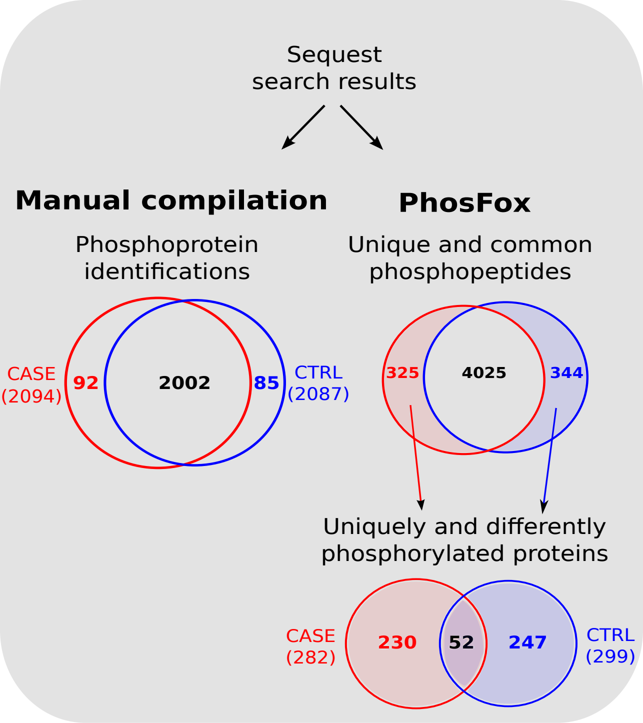
Sequest
search results
Manual compilation
Phosphoprotein
identifications
CASE
(2094)
92
2002
85
CTRL
(2087)
PhosFox
Unique and common
phosphopeptides
325
4025
344
Uniquely and differently
phosphorylated proteins
CASE
(282)
230
52
247
CTRL
(299)

Figure S1
